# Supplementary material for: Allele Specific Expression Quality Control Fills Critical Gap in Transcriptome Assisted Rare Variant Interpretation
Source: bioRxiv. 2025 Jun 8:2025.05.30.657086. Preprint. [Version 2] doi: 10.1101/2025.05.30.657086 (PMC12157414; doi:10.1101/2025.05.30.657086)
Supplement: Supplement 1 [file media-1.zip › Supp_Text_Fixed_Ref_June_7.pdf]

Allele Specific Expression Quality Control Fills Critical Gap in Transcriptome Assisted Rare Variant Interpretation

Kaushik Ram Ganapathy[1, 2], Eric Song[1], Daniel Munro[3, 4], Ali Torkamani[1, 2], Pejman Mohammadi[1, 3, 5 *]

[1] Dept. of Integrative Structural and Computational Biology, Scripps Research, La Jolla, CA, USA

[2] Scripps Research Translational Institute, La Jolla, CA, USA

[3] Center for Immunity and Immunotherapies, Seattle Children's Research Institute, Seattle, WA, USA

[4] Dept. of Psychiatry, University of California San Diego, La Jolla, CA, USA

[5] Department of Pediatrics, University of Washington School of Medicine, Seattle, WA, USA *

Corresponding Author: pejmanm@uw.edu

Supplementary Images

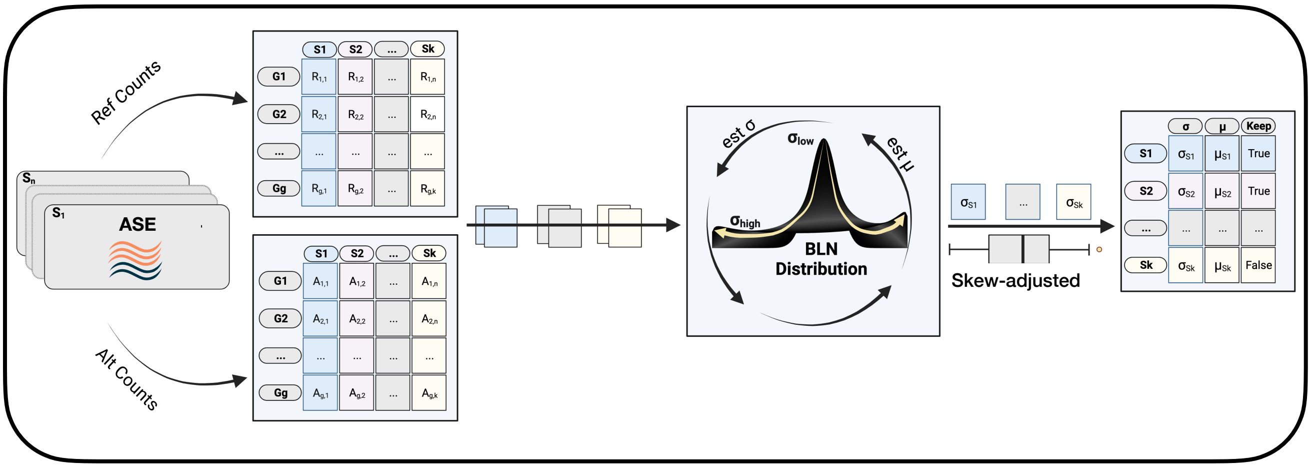

Figure S1: Workflow to Quantify Quality in Sample-Level ASE Data Using BLN Distribution
Schematic representation of the aseQC pipeline for cohorts, detailing the calculation of σ for each sample with the application of a skew-adjusted boxplot to establish quality thresholds [1]

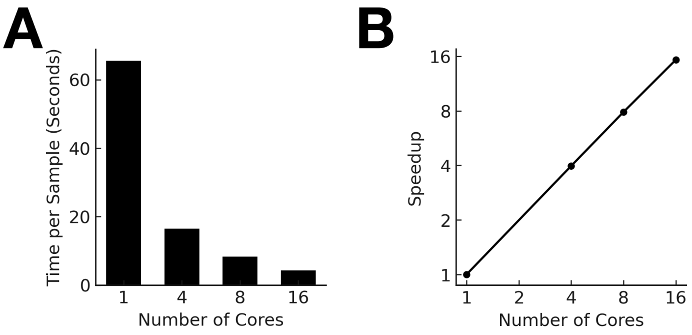

Figure S2: Runtime Evaluation of aseQC

(A) Per-sample runtimes for 64 identical ASE samples, showing the effect of varying the number of cores on aseQC fit performance. **(B)** Speed-up achieved by aseQC relative to single-core performance as the number of cores increases.

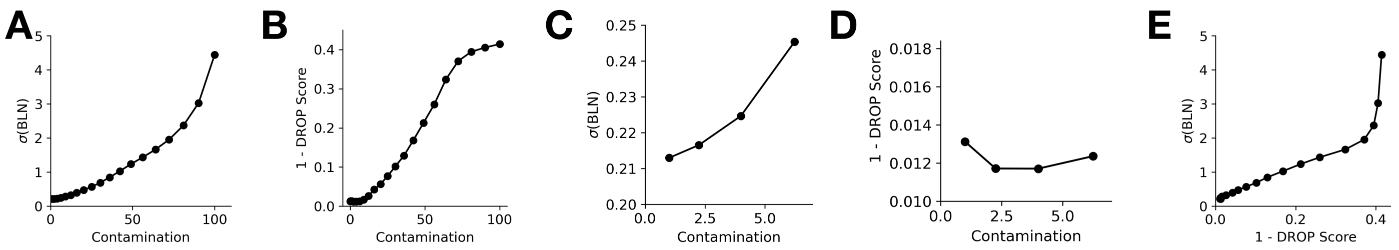

Figure S3: Comparing Detection of Sample Mismatches by aseQC and DROP-MAE for Sample HG00139
(A) aseQC shows a monotonically increasing σ(BLN) with rising contamination. **(B)** DROP-MAE QC exhibits a sigmoid response curve, plateauing at higher contamination levels. **(C)** aseQC is more responsive particularly at higher contamination levels.

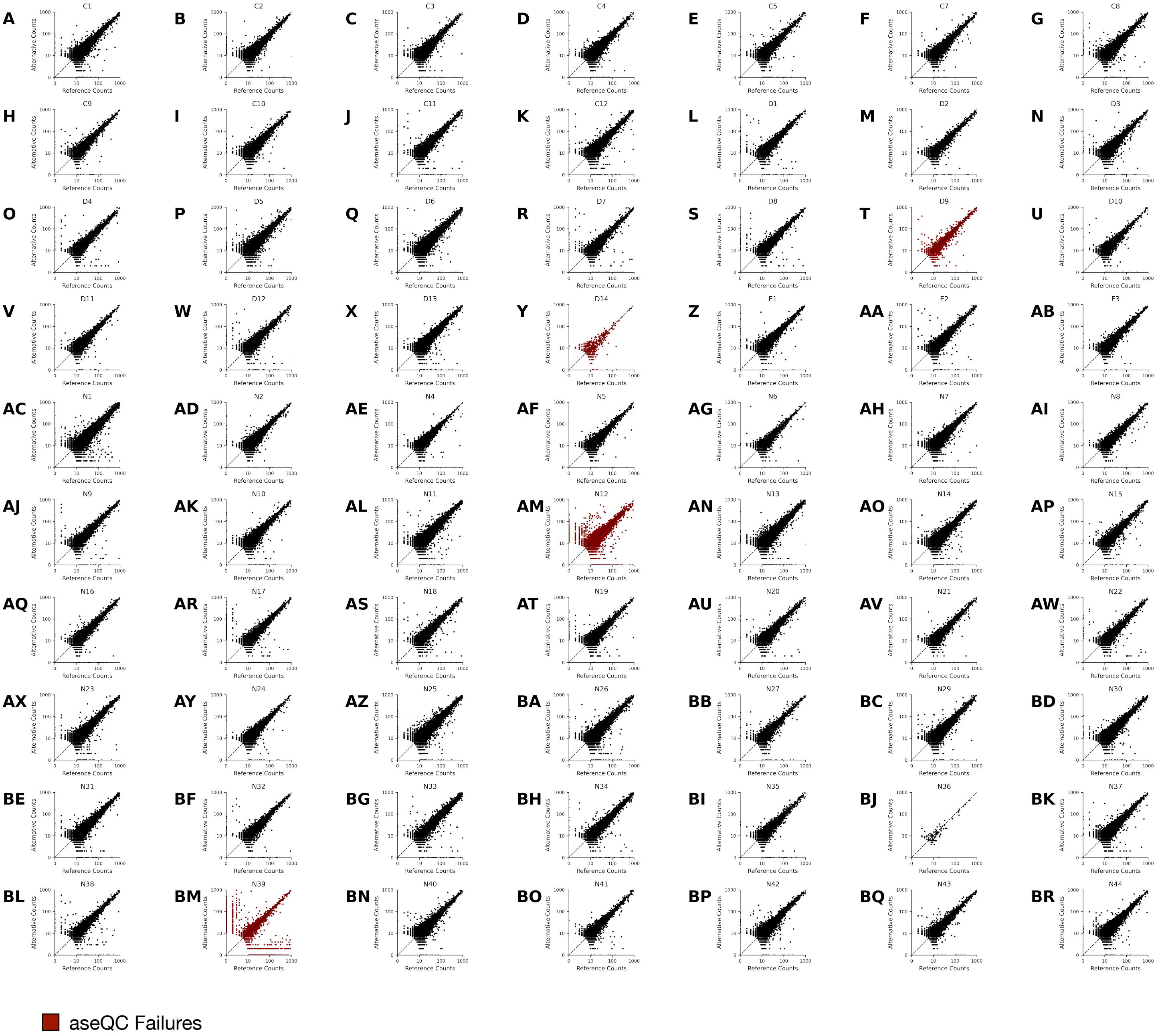

Figure S4: Applying aseQC to a Cohort of 70 Muscular Dystrophy Cases

Applying aseQC identifies 4 samples (in red) that failed the aseQC test. 2 of these samples (panels BM, AM) are known previously to be of low quality.

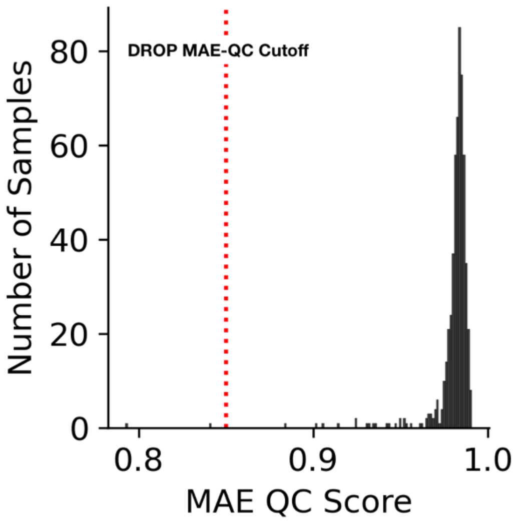

Figure S5: Comparison of DROP MAE QC scores among Samples Failing aseQC
Histogram depicting DROP MAE-QC scores for 563 samples that failed aseQC. The dotted red line represents the DROP MAE-QC passing threshold. Notably, all but two aseQC-failing samples meet the DROP MAE-QC criteria suggesting no genotype-RNA mismatches.

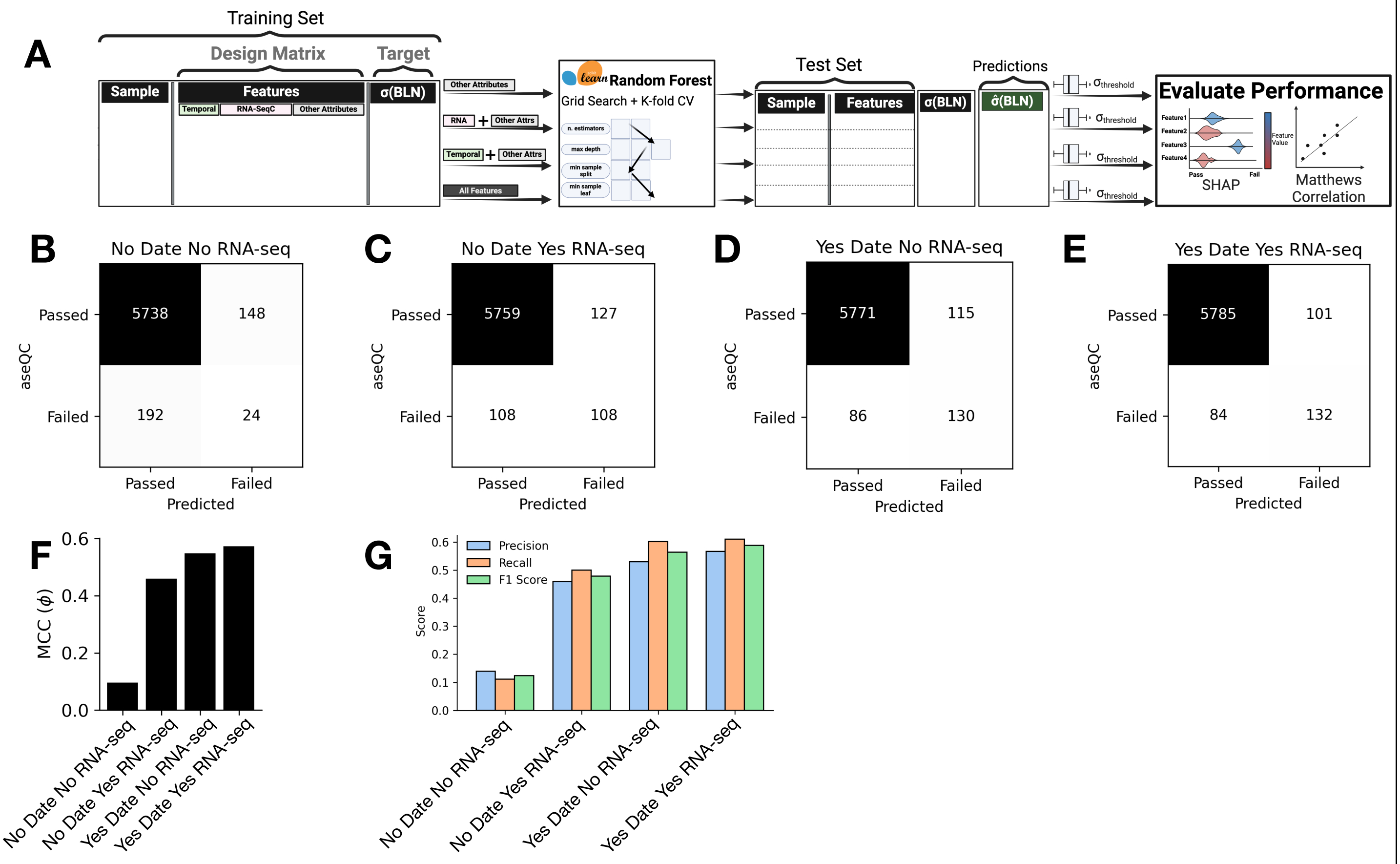

Figure S6: Evaluation of Random Forest Models for Predicting aseQC score from Sample Attributes
(A) Schematic workflow illustrating the Random Forest model fitting process to identify attributes linked to ASE sample quality [2]. **(B-E)** Confusion matrices depicting prediction performance on the test set across four models with different feature sets. **(F)** Matthew's Correlation Coefficient (MCC (ϕ); r-score) for the test set across the four models. **(G)** Precision, Recall, and F1 scores for all four models on the test set.

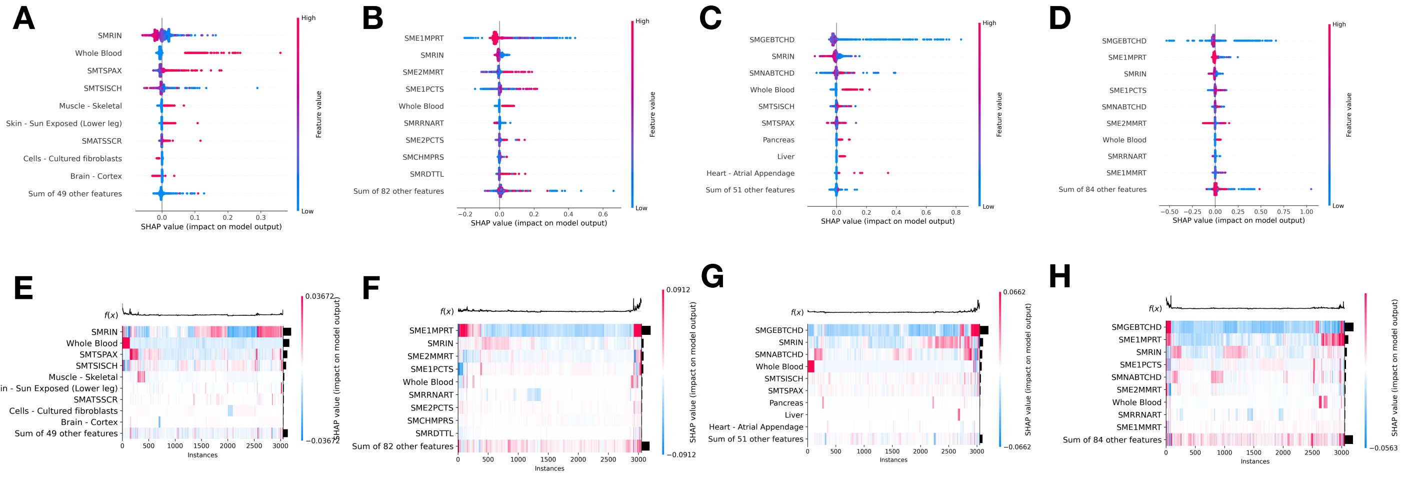

Figure S7: SHAP Plots Highlighting Features Contributing to aseQC Score Prediction
(A-D) Beeswarm plots illustrating how feature values influence SHAP scores, driving predictions toward lower values (aseQC pass) or higher values (aseQC failure). **(E-H)** Heatmaps depicting the interactions among features, showcasing their combined impact on predicting aseQC passes or failures across all four models.

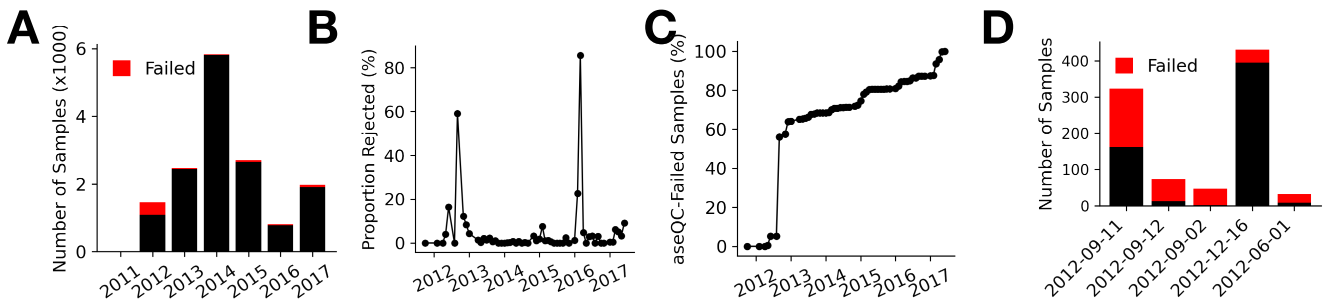

Figure S8: aseQC Failures by Sample Processing Date
(A) Number of aseQC Failures aggregated per year **(B)** Proportion of aseQC sample failures aggregated by month. **(C)** Cumulative distribution of the proportion of total samples failed. **(D)** Top 5 days with the highest number of aseQC failures.

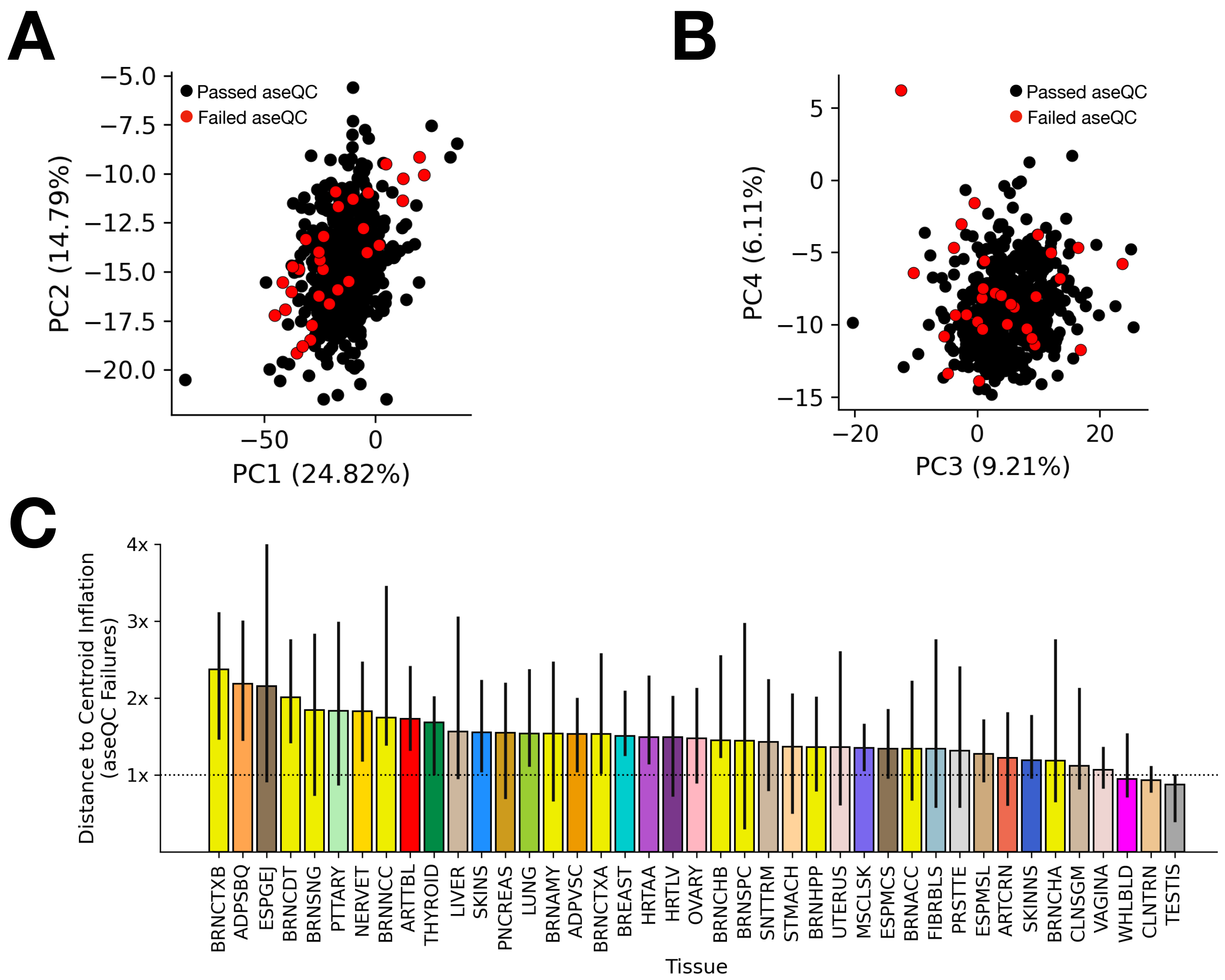

Figure S9: PCA-Based Assessment of Global Gene Expression Deviation in aseQC-Failed Samples Across GTEx Tissues.

(A) PC1 vs PC2 and **(B)** PC3 vs PC4 projections of log-transformed gene expression profiles for Adipose Subcutaneous tissue, with samples colored by aseQC status (black = passing, red = failing). **(C)** Fold-change in median Euclidean distance to the tissue-specific centroid, defined as the average position of all samples in four-dimensional PCA space, comparing aseQC-failed to passing samples across GTEx tissues with >25 samples and at least 5 failures. Error bars represent 95% bootstrap confidence intervals.

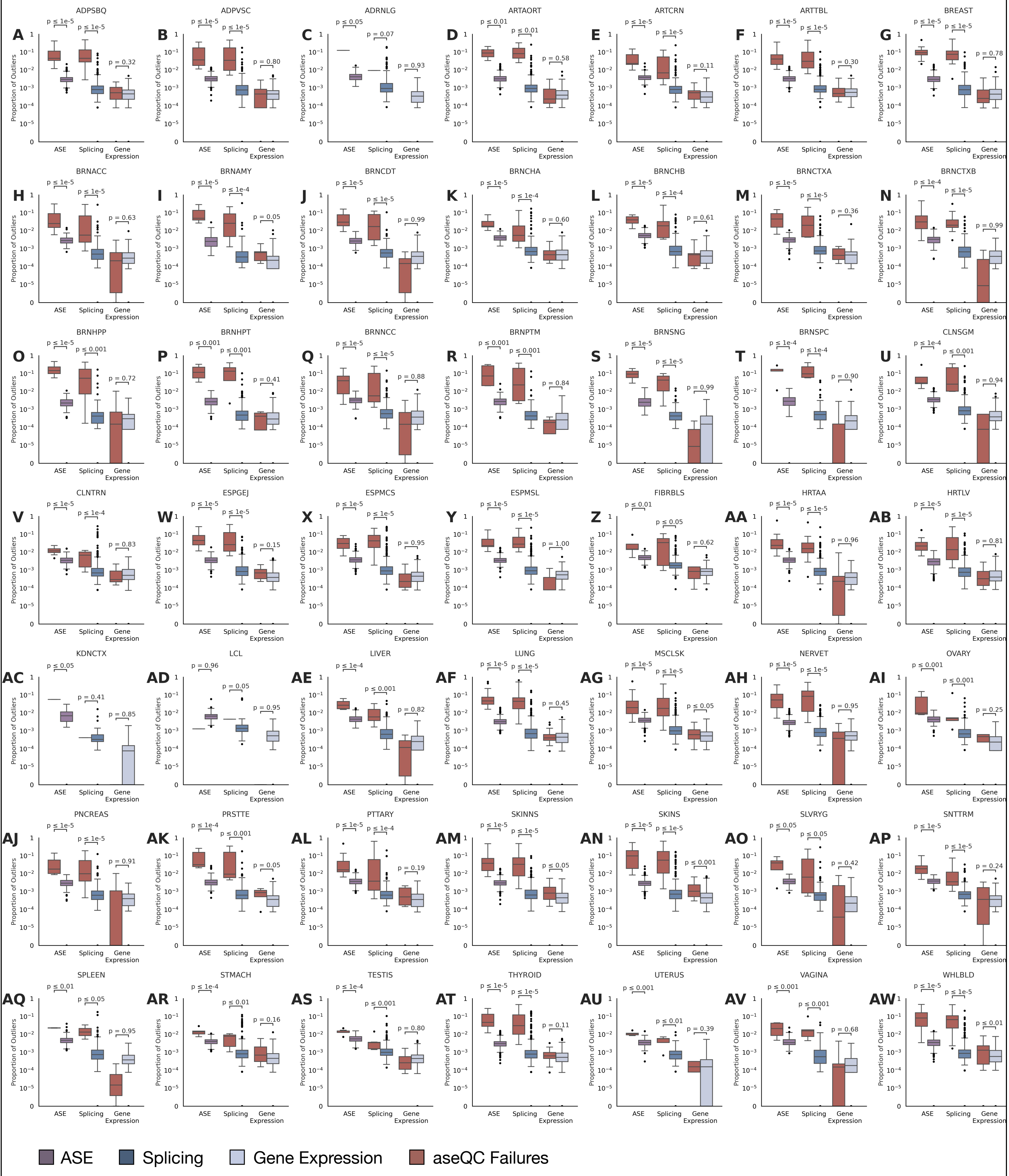

Figure S10: Proportion of outlying genes identified in samples categorized as passed or failed by aseQC. (A-AW) Outlying genes were detected using ANEVA-DOT for allele-specific expression (ASE), FRASER2 for splicing outliers, and OutSingle for gene expression outliers for all GTEx tissues with over 25 samples.

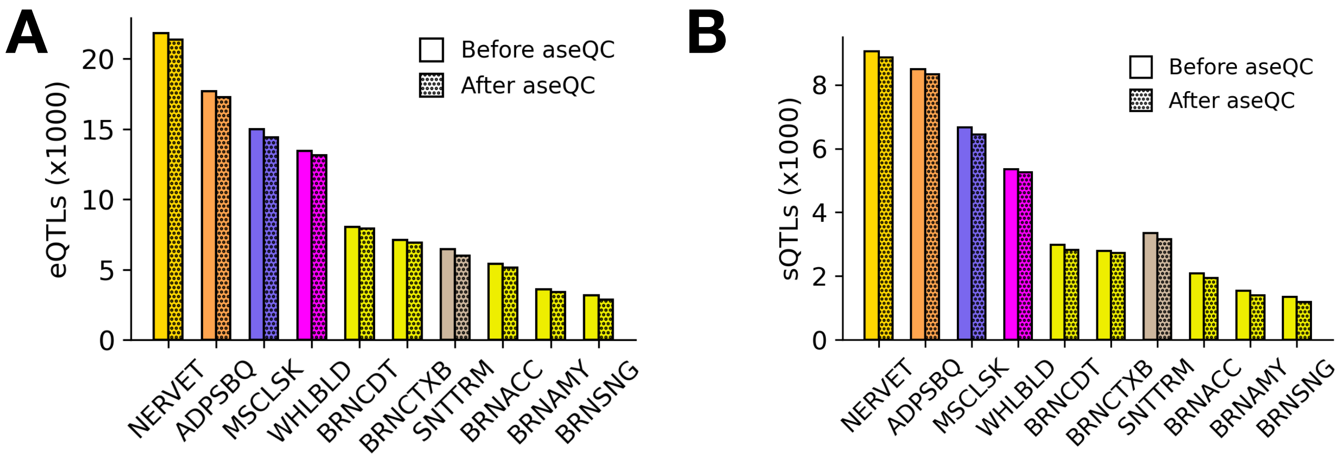

Figure S11: Effect of aseQC-based filtering on cis-QTL discovery in GTEx tissues.
(A) Number of significant eQTLs and **(B)** sQTLs identified before and after excluding aseQC-failed samples, shown for the top-ten GTEx tissues (n > 25) with most proportion of aseQC failures

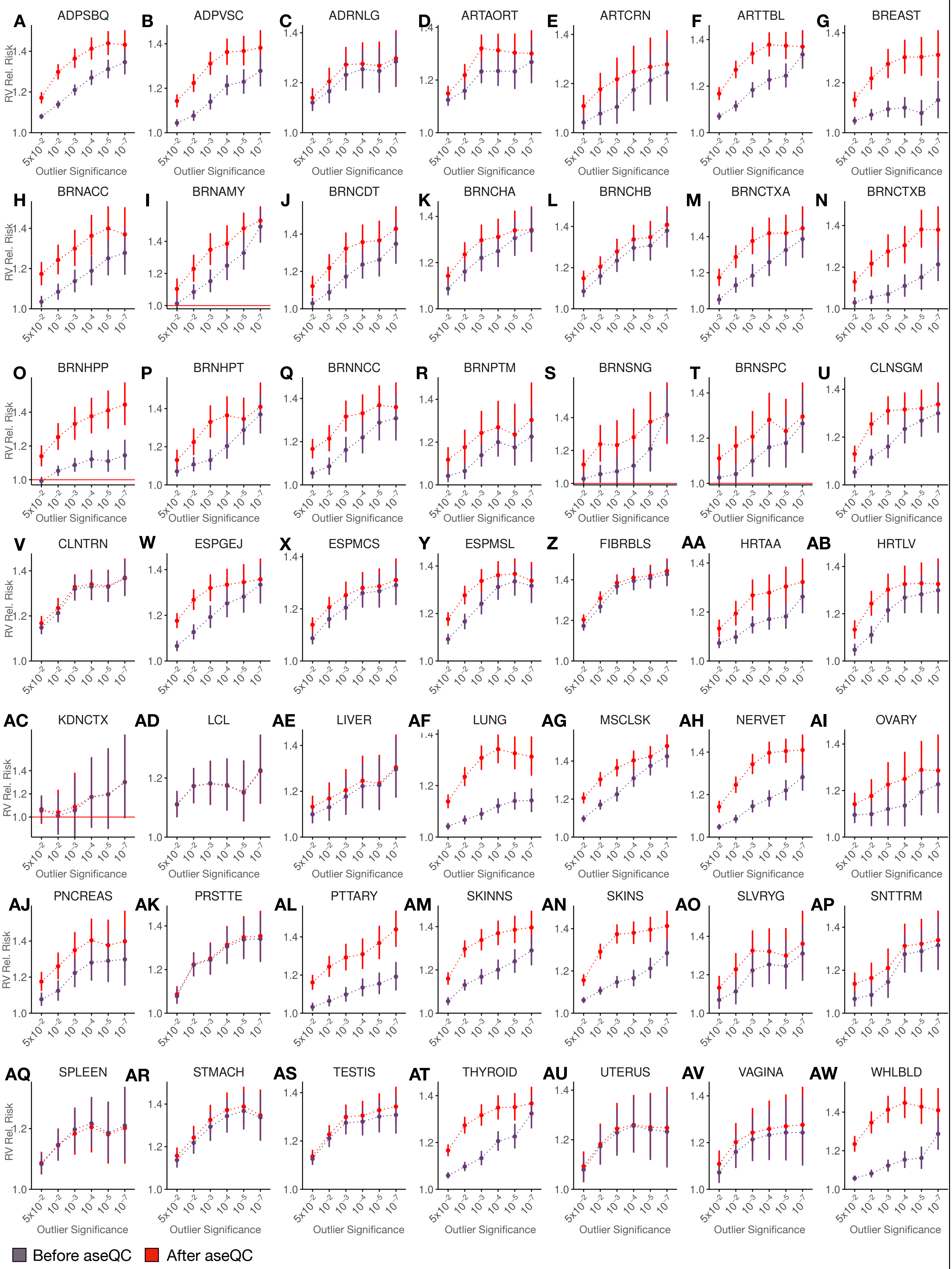

Figure S12: Changes to ASE Rare Variant Enrichment by Outlier Significance Post-aseQC Filtering (A-AW) Comparison of changes to rare variant enrichment stratified by DOT outlier significance after removing aseQC failures in 49 GTEx tissues.

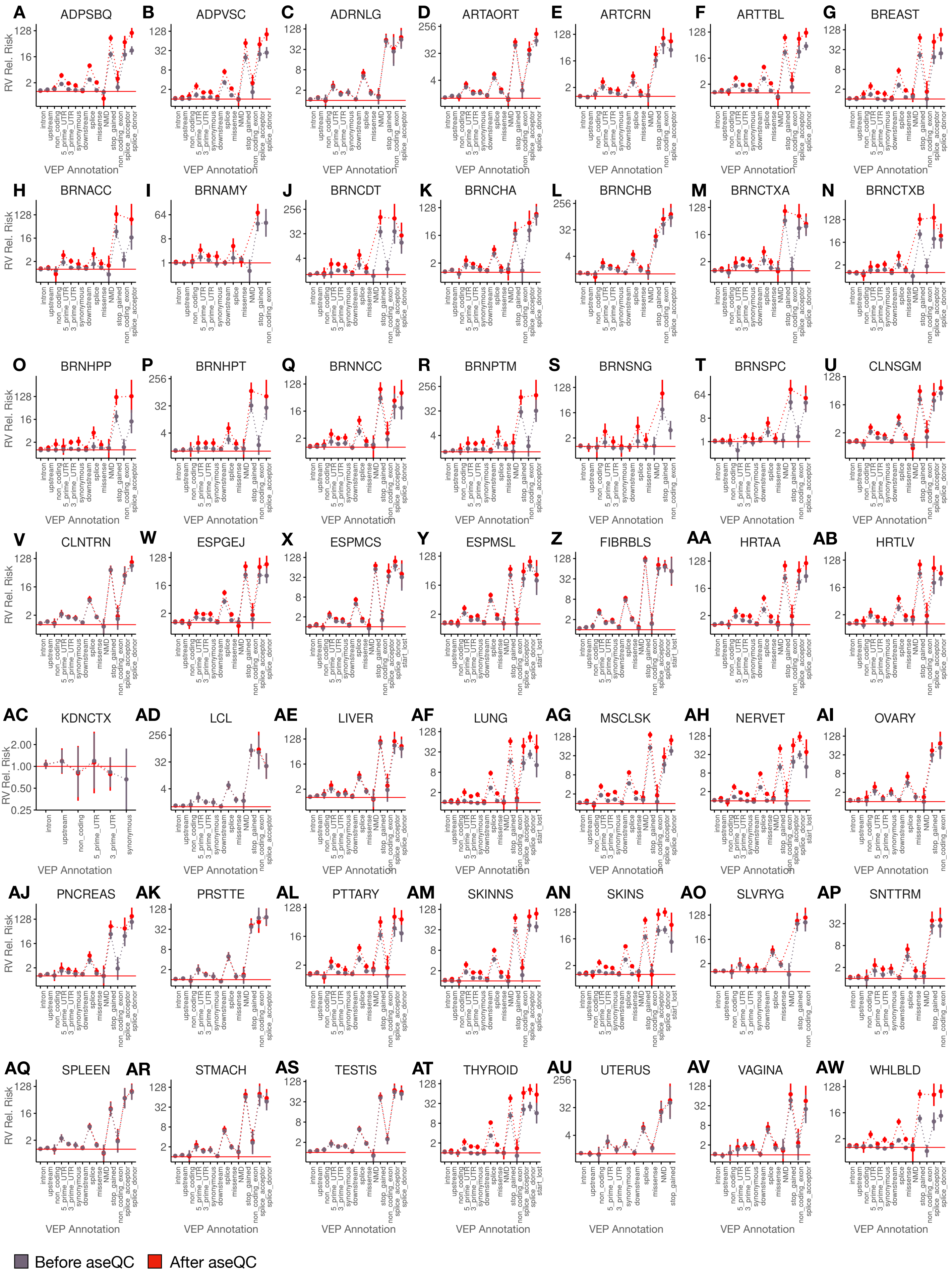

Figure S13: Changes to ASE Rare Variant Enrichment by VEP Consequence Post-aseQC Filtering

(A-AW) Comparison of changes to rare variant enrichment among DOT outliers (FDR qval <= 0.05) stratified by VEP consequence in variant- and haplotype-aggregated ASE data in 49 GTEx tissues.

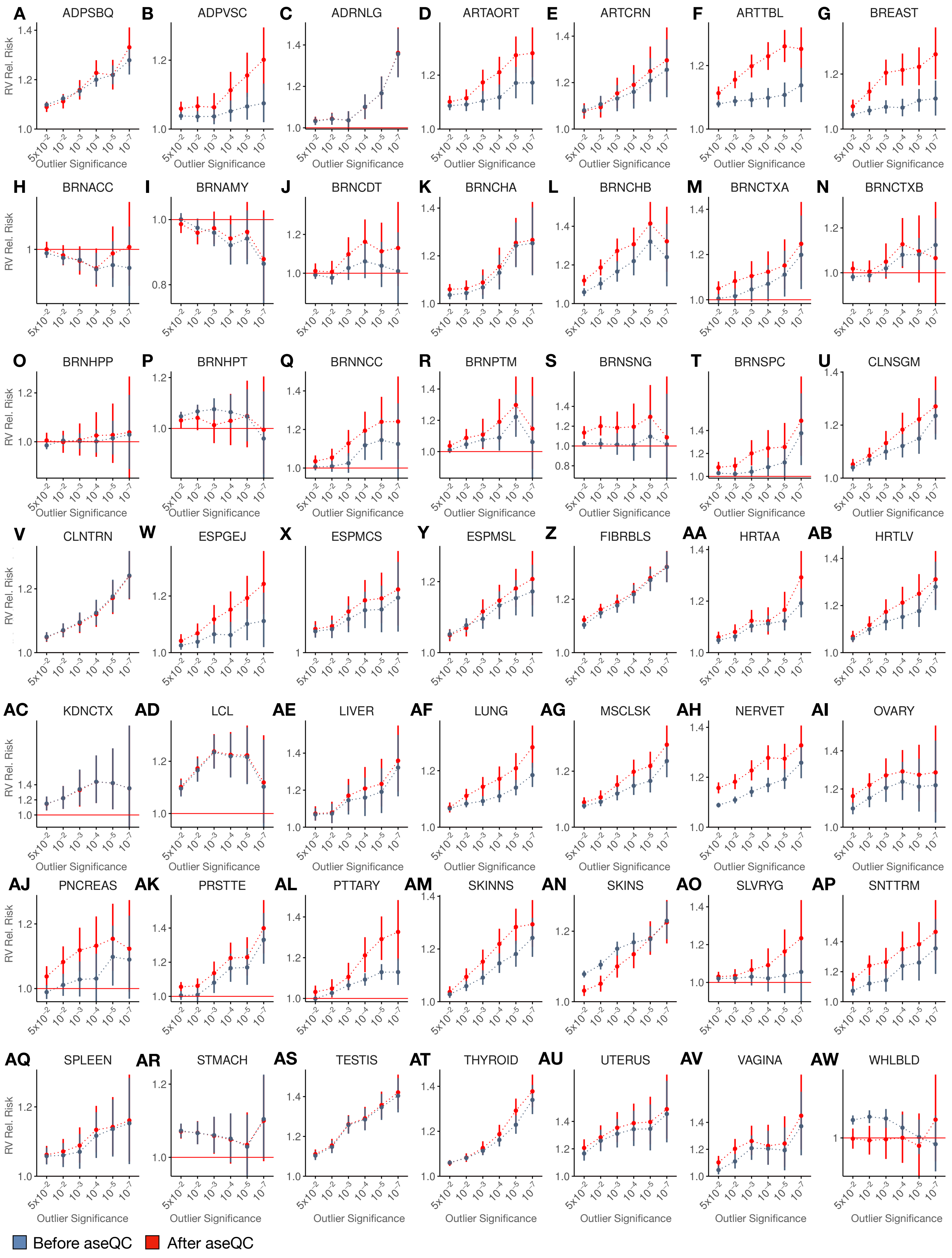

Figure S14: Changes to Splicing Rare Variant Enrichment by Outlier Significance Post-aseQC Filtering (A-AW) Comparison of changes to rare variant enrichment stratified by FRASER2 outlier significance after removing aseQC failures in 49 GTEx tissues.

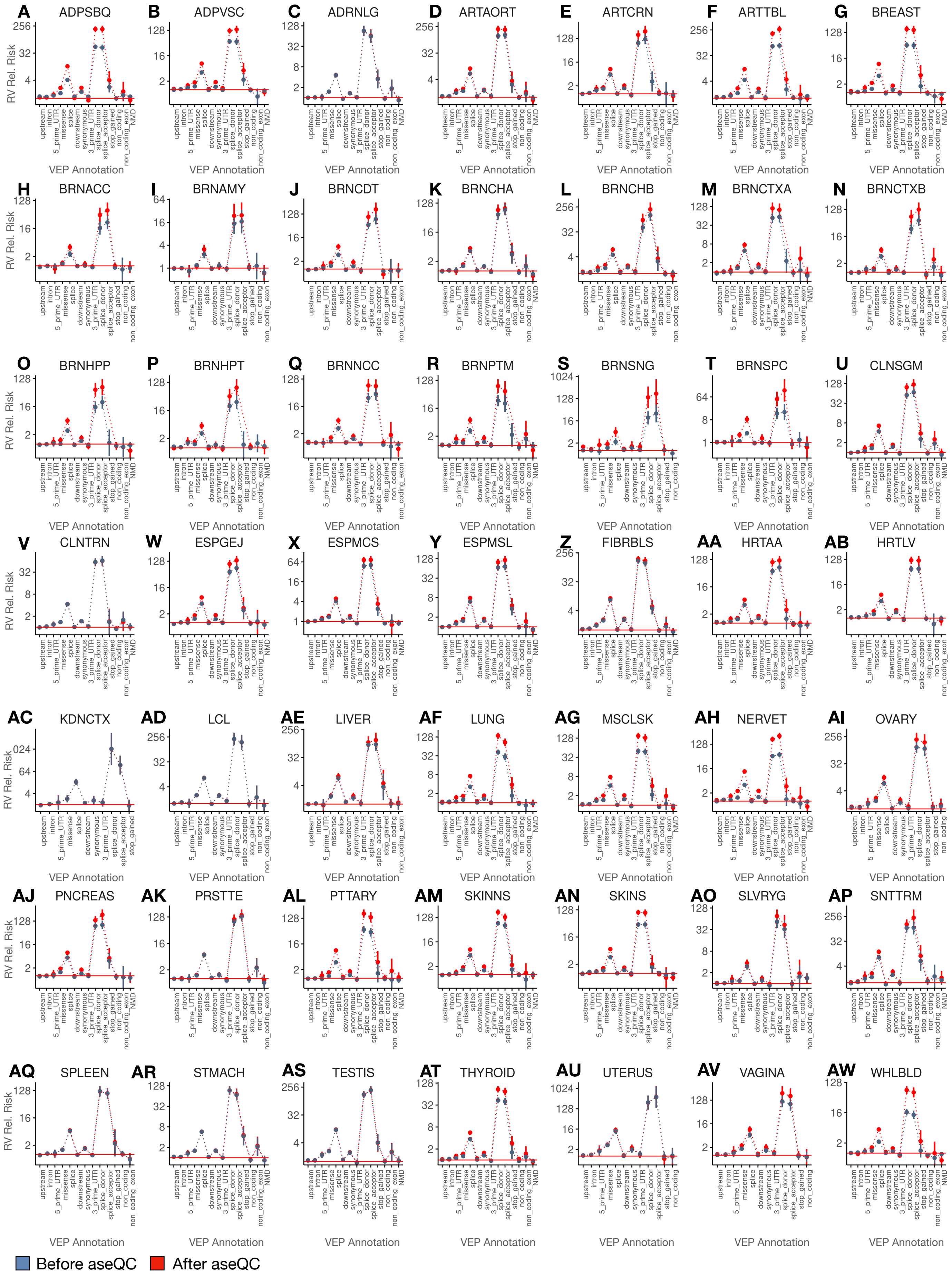

Figure S15: Changes to Splicing Rare Variant Enrichment by VEP Consequence Post-aseQC Filtering (A-AW) Comparison of changes to rare variant enrichment among FRASER2 outliers (FDR qval <= 0.05) stratified by VEP consequence in variant- and haplotype-aggregated ASE data in 49 GTEx tissues.

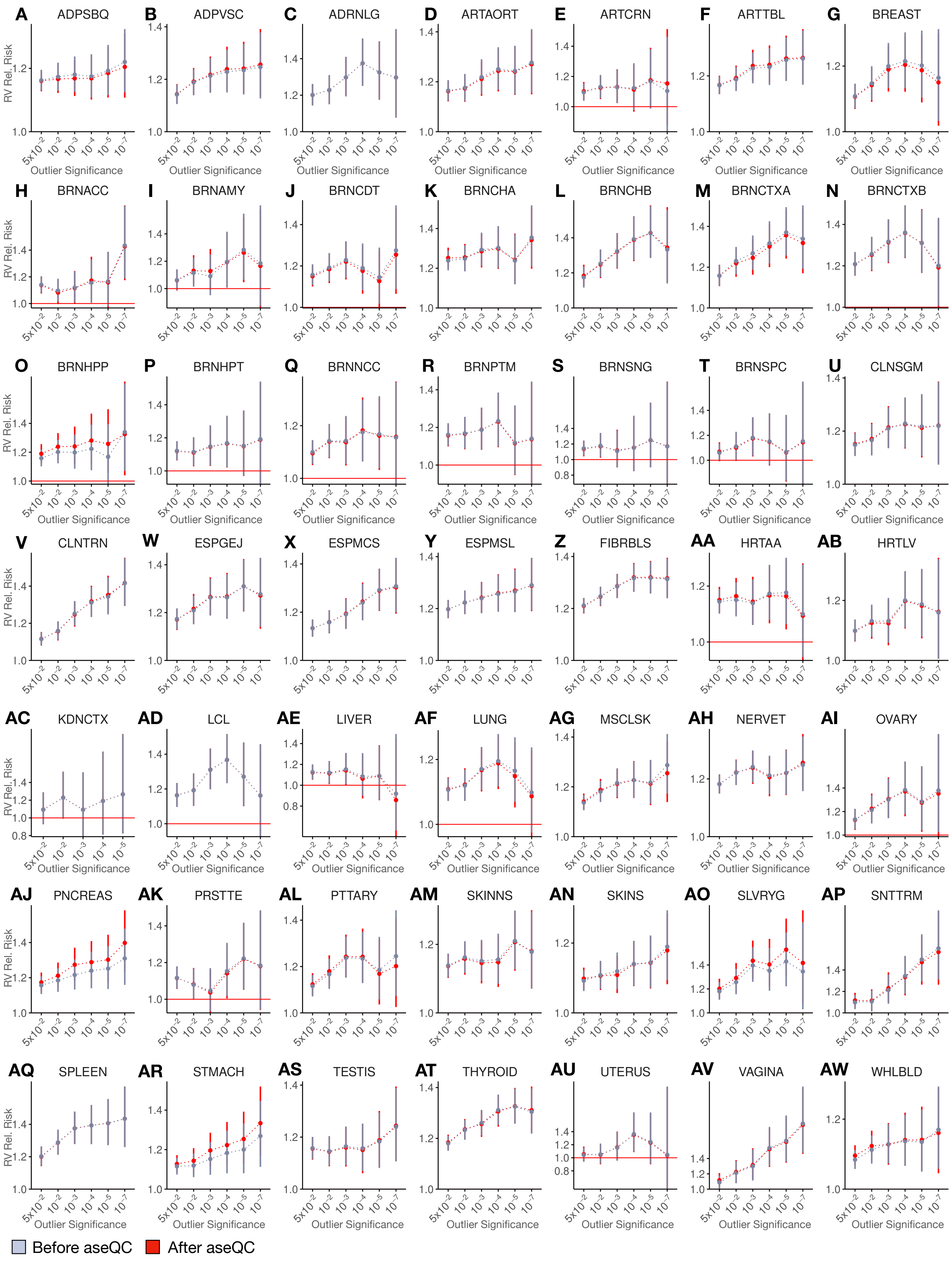

Figure S16: Changes to Gene Expression Rare Variant Enrichment by Outlier Significance Post-aseQC Filtering

(A-AW) Comparison of changes to rare variant enrichment stratified by OutSingle outlier significance after removing aseQC failures in 49 GTEx tissues.

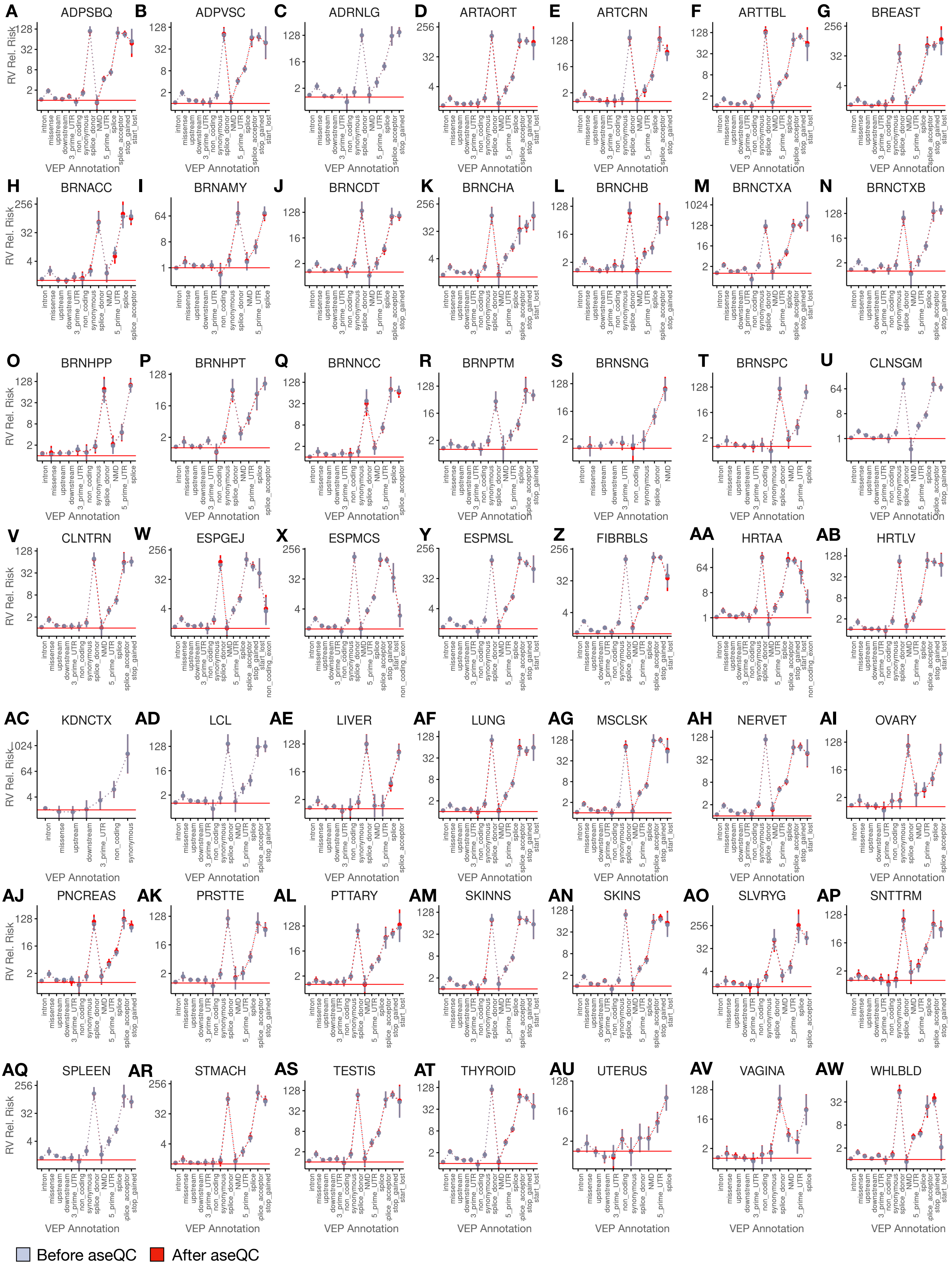

Figure S17: Changes to Gene Expression Rare Variant Enrichment by VEP Consequence Post-aseQC Filtering

(A-AW) Comparison of changes to rare variant enrichment among OutSingle outliers (FDR qval <= 0.05) stratified by VEP consequence in variant- and haplotype-aggregated ASE data in 49 GTEx tissues.

References

1. Ganapathy, K. *Quantifying Quality in Sample-Level ASE Data Using BLN Distribution*. [2025] 2025; Available from: https://BioRender.com/kyfvgca.
2. Ganapathy, K. *Evaluation of Random Forest Models for Predicting aseQC Score from Sample Attributes*. [2025] 2025; Available from: https://biorender.com/7r0s8ue.
